# A Single Versatile Appliance for Habit Interception and Crossbite Correction

**DOI:** 10.1155/2015/607545

**Published:** 2015-11-12

**Authors:** Mohammed Zameer, Syed Nahid Basheer, Arun Reddy, Suresh Kumar Kovvuru

**Affiliations:** ^1^Department of Pediatric & Preventive Dentistry, Navodaya Dental College and Hospital, Rajiv Gandhi University of Health Sciences, Raichur, Karnataka 584103, India; ^2^Department of Restorative Dental Science, College of Dentistry, Jazan University, Jazan 45142, Saudi Arabia; ^3^Department of Orthodontics, Navodaya Dental College and Hospital, Rajiv Gandhi University of Health Sciences, Raichur, Karnataka 584103, India; ^4^Department of Conservative Dentistry & Endodontics, KVG Dental College & Hospital, Rajiv Gandhi University of Health Sciences, Sullia, Karnataka 574239, India

## Abstract

Digit sucking is a common childhood behavior, which has an adaptive value for children up to the fourth year of life. It is usually associated with oral pleasure and self-comforting behavior. But chronic practice may produce deleterious effect in the form of dental and skeletal deformities. Adjunctive therapy using bluegrass appliance as a permanent reminder and quadhelix appliance as a reminder as well as a slow palatal expander has proven successful in intercepting digit-sucking habit and expanding the arch for crossbite correction. In the present case, a versatile modified quadhelix appliance incorporating a roller was designed to clinically correct the habit and its resulting dentofacial deformities.

## 1. Introduction

Many children suck their thumbs or fingers for short periods during infancy or early childhood. The habit may be considered normal during the first 2 years of life. If it gradually diminishes, the child probably will stop the habit. On the other hand, if the habit persists or increases in frequency then adverse dental and skeletal changes are noted [1].

The persistent, active thumb sucking habit frequently results in expression of dentofacial deformities. These include (1) anterior open bite, (2) increased overjet, (3) lingual inclination lower incisor and labial inclination upper incisor, (4) posterior crossbite, (5) compensatory tongue thrust, (6) deep palate, (7) speech defect, and (8) finger defects (eczema of the finger due to alternate dryness and moisture that occurs and even angulations of the finger) [2–6].

Pathway of care for interception of habit and correction of these deformities often requires multiple appliances which can lead to increase of the treatment time and cost. Therefore, the surrogate in the need of a single, convenient, expeditious, and cost-effective appliance was devised as a combination or single versatile appliance in the present case report to simultaneously intercept the practice of sucking habit and correct the orofacial and dental abnormalities.

## 2. Case Presentation

An 11-year-old girl accompanied by her mother was reported to the Department of Pediatric and Preventive Dentistry, Navodaya Dental College and Hospital. The chief complaint was narrated by the mother describing the unaware daytime and nocturnal thumb sucking habit of her daughter till the present age. An appropriate habit history and clinical evaluation was done along with the patient pretreatment record including radiographs, study models, and photographs.

### 2.1. Habit History

It revealed that she was the 3rd child to her parents who belongs to a middle class family. The other two elder siblings also had the habit. Her relationship with her peers was good but she was subjected to being cursed upon practicing the habit. Psychological evaluation revealed positive attitude towards discontinuing the habit practice.

### 2.2. Clinical Examination (Figures 1 and 2)

Extraorally, it revealed depression just above the corner of the mouth, and the lips were slightly inwards at the corner of the mouth. Intraorally, there were anterior open bite of 2 mm, proclined upper/lower incisors except left lateral incisor which was palatally locked, unilateral posterior crossbite involving the two premolars with constricted maxilla on the left, compensatory tongue thrust, and mild lisping in her speech.

### 2.3. Treatment Plan

A brief treatment plan was formulated comprising initially parent and patient counseling. This was done by discussing the problems or the deleterious effects of a persistent sucking habit with the audiovisual aids. Patient responded positively and was motivated to stop practicing the habit. Parents were also counseled to motivate and support her in discontinuing the habit.

**Figure 1:**
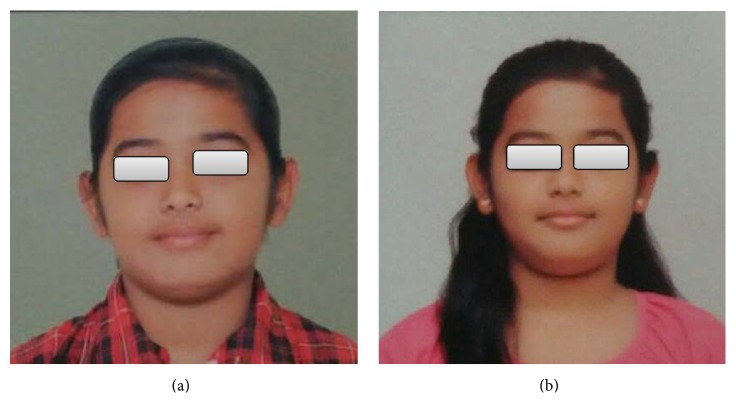
Facial images. (a) Depression just above the corner of the mouth and the lips were slightly inward at the corner of the mouth. (b) Deformities were refrained after crossbite correction.

**Figure 3:**
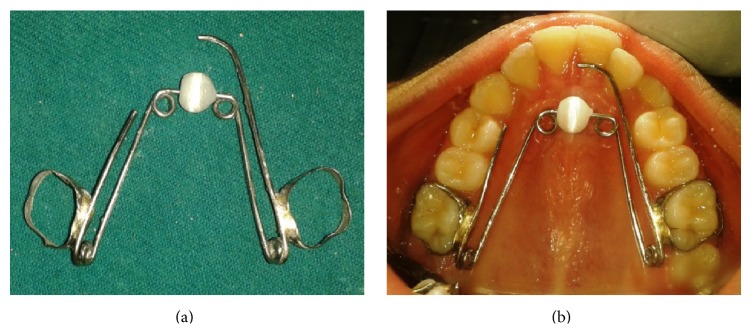
Design of modified quadhelix appliance with a roller. (a) Construction. (b) Insertion of the appliance.

**Figure 4:**
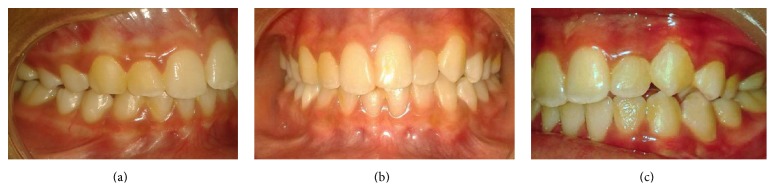
Postoperative condition indicating correction of (a) anterior open bite, (b) posterior crossbite, and (c) alignment of the palatally locked lateral incisor.

**Figure 5:**
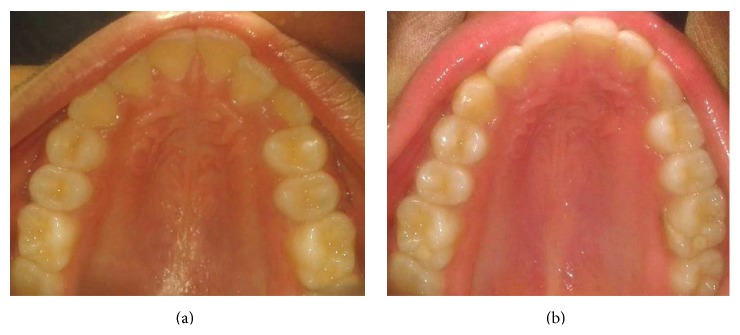
Pre- and postoperative occlusal view.

She was started on the modified quadhelix appliance with a roller (Figure 3). During this she showed positive attitude and was willing to try the appliance as an aid to stop the habit. She was on a passive appliance initially followed by activation of the appliance to correct the deformities for a total period of 9 months. At the end of the treatment, she intercepted the habit practice and the deformities were refrained and transformed to a proper relationship (Figures 1, 4, and 5). There was correction of anterior open bite and correction of posterior crossbite along with the alignment of the palatally locked incisor.

## 3. Appliance Design

A modified quadhelix appliance was constructed using a 0.036-inch stainless wire incorporating an acrylic roller. The roller was added to the anterior arm as a reminder and from posterior arm an anterior extension was made to bring the lateral incisor into alignment along with crossbite correction. The palatal wire was made contacting the teeth in crossbite and palatally placed lateral incisor anteriorly. It was kept 1 to 1.5 mm away from the marginal gingiva and palatal tissue and extended 1-2 mm posteriorly to the banded molars to eliminate soft tissue irritation. The wire component with the roller was soldered to the bands on permanent first molars.

## 4. Appliance Placement and Clinical Management

The use of habit-breaking appliance was discussed with parents and the child, with a thorough explanation of the purpose of appliance. It was indicated that the patient wanted to stop the habit and was also willing to try the appliance as an aid to stop. Treatment initiated with orthodontic separators, followed by try-in to ensure the optimal fit of appliance. The whole appliance was then cemented using glass ionomer cement (GC Fuji 1). Treatment duration was planned to be 6–9 months up to 1 year. The patient and parent were informed that there may be some discomfort as the child becomes accustomed to the appliance and deals with the inability to suck digits. The roller in this modified quadhelix appliance was left in place and continuously monitored and removed in six months after correcting the thumb sucking and compensatory tongue thrust habit.

Expansion of the appliance was started in the second month after its insertion to allow the child to acclimatize to the new appliance and deal with the habit-breaking aspect initially. Expansion was carried out selectively in the posterior arm leaving the anterior arm with the roller. The anterior open bite was found to be corrected spontaneously following cessation of the habit. The unilateral posterior crossbite (left) was corrected by incremental activation of the appliance. The crossbite correction was monitored and the appliance was reactivated until overcorrection was achieved which took approximately 3 months. The appliance was then maintained for further two to three months to retain the correction in the coronal plane. Some consequences from the use of this appliance, as with any orthodontic appliance, might include tongue irritation, temporary discomfort, speech and eating difficulties, breakages, and difficulty with oral hygiene. Patient was educated about these in advance.

## 5. Discussion

Clinical management of sucking habit through conventional therapies includes sequential use of multiple appliances to correct each of the problems individually. This will definitely lead to increase treatment period as well as additional expenses. Thus, the single versatile appliance introduced in the presented case report has reduced the treatment duration as well as the cost and corrected the (1) thumb sucking habit, (2) anterior open bite, (3) unilateral posterior crossbite, and (4) compensatory tongue thrust habit without the need of multiple appliance strategies. Habit-breaking therapy using crib-rake type appliances has been argued and regarded as a punitive rather than a supportive treatment [7]. Thus, an appropriate, nonthreatening, positive reinforcing approach as modified quadhelix appliance with a roller was used in the presented case report.

Giuntini et al. [8] have introduced a single appliance consisting of a quadhelix with the addition of a crib. It has shown to be effective, producing favorable dental effects and patient compliance. The modified quadhelix appliance with a roller has ruled out the disadvantages of crib system. The roller is placed in the most superior aspect of the palate which does not cause obstruction with eating, emotional problems, and iatrogenically “self-inflicted” wounds and presents minimal disturbance to speech. The roller of the appliance is based on the principles of positive reinforcement which works through a counterconditioning response to the original conditioned stimulus for thumb sucking. That is, the child can be instructed to play by spinning the roller with tongue which establishes a new nonharmful habit of playing with the roller [9]. Literature has proved the bluegrass appliance was comfortable to the patients [9–12] and shown its effectiveness in cessation of thumb sucking habit within a short period of time [9–13]. The helices of the quadhelix appliance itself serve to remind the child not to place the finger in the mouth [14]. Additionally, the roller in the modified quadhelix design gives a synergistic effect in reminding the child for the interception of sucking habit.

Haryett et al. [15] suggested that the roller in the habit-breaking appliance be placed for six months after the cessation of habit to ensure that the habit should not resume. Thus, six-month continuation of the appliance was followed in the presented case. Long-term familiarity with the roller has also been shown to reduce the oral gratification and dependency upon appliance use. This has ultimately resulted in the elimination of digit-sucking habit and the dependency upon a positive reinforce was slowly removed.

Correction of the unilateral posterior crossbite was achieved by progressive unilateral activation (expansion) of the modified quadhelix appliance laterally using a 3-prong plier. The anterior extension of the outer arm of the quadhelix appliance has brought the palatally placed lateral incisor into alignment. Thus, modification of the quadhelix with an anterior extension can work simultaneously for crossbite correction and anterior teeth alignment.

Proclination of the maxillary anterior teeth was spontaneously corrected following cessation of the habit. This was achieved by alleviation of the labial forces through habit practice and allowance of the normal labial pressure exertion from the lip. Although the modified quadhelix design with a roller has given versatile characteristics, it has few limitations. Until the roller is removed, that is, usually six months after interception of the habit, it can be applicable only to correct the teeth in crossbite which are anterior to the molar which was indicated in our case. It is because the anterior bridge is incorporated with a roller which would not allow its activation. To overcome this, an accessory wire in front of the anterior bridge can be constructed incorporated with a roller which will not hinder the activation of the appliance at the anterior bridge. Another limitation is that to a lesser degree oral hygiene maintenance with quadhelix helix appliance is difficult. Patient should be informed and reinforced for proper oral hygiene maintenance.

## 6. Conclusion

This modified quadhelix design with a roller can be considered as an optional treatment strategy for simultaneous interception of sucking habit and correction of its resulting dentofacial deformities.

## Figures and Tables

**Figure 2:**
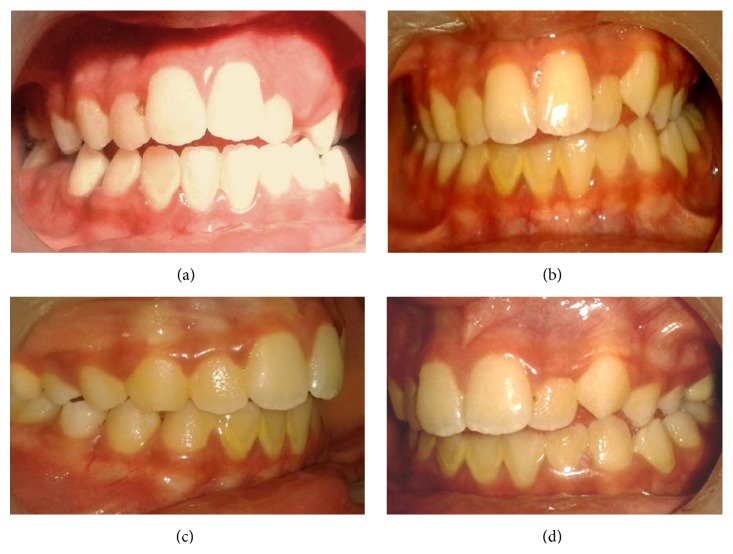
Preoperative condition indicates (a) anterior open bite, (b) compensatory tongue thrust, and ((c) and (d)) unilateral (left) posterior crossbite and palatally locked lateral incisor.
